# Investigating the effects of Citrullus colocynthis pulp on oxidative stress in testes and epididymis in streptozotocin-induced diabetic male rats

**Published:** 2017-01

**Authors:** Fereshteh Ostovan, Ali Gol, Abdolreza Javadi

**Affiliations:** 1 *Faculty of Science, Payam noor University, Iranshhar, Iran.*; 2 *Department of Biology, Faculty of Science, University of Shahid Bahonar, Kerman, Iran.*; 3 *Pathology Department, Beheshti University of Medical Sciences, Tehran, Iran.*

**Keywords:** Diabetes, Catalase, Peroxidase, Malondialdehyde, Hydrogen peroxide, Oxidant

## Abstract

**Background:**

Diabetes mellitus is one of the most common metabolic diseases in humans, affecting 100 million people around the world.

**Objective:**

Investigating the effects of Citrullus colocynthis pulp on oxidant and antioxidant factors of testes and epididymis in streptozotocin-induced diabetic male rats.

**Materials and Methods:**

Thirty two male rats were divided into four groups (n=8) 1) N (normal) group, 2) N+C group, 3) D (diabetic) group and 4) D+C group. Groups N and D received normal saline 2 ml orally for two weeks and groups N+C and D+C received 10 mg/kg.bw Citrullus colocynthis pulp orally for two weeks. Diabetes was induced by single intraperitoneal injection of streptozotocin (STZ) at 65 mg/kg.

**Results:**

D group had a significant increase in H_2_O_2_ (Hydrogen peroxide) and MDA (malondialdehyde) concentrations, and CAT (catalase) activity, and also a significant decrease in Peroxidase (POD) activity compared to N group. D+C group had a significant decrease in H_2_O_2_ and MDA concentrations and, CAT activity and significant increase in POD activity compared to D group.

**Conclusion:**

Citrullus colocynthis pulp in two weeks had beneficial effects on oxidants and antioxidants changes in reproductive system in streptozotocin-induced diabetic rats.

## Introduction

Diabetes mellitus (DM) is one of the most common metabolic diseases in humans, affecting 100 million people around the world (1). In the human, DM is thought to occur in two different ways. Type I diabetes is caused by autoimmume destruction of insulin producing beta-cells of the pancreas, and commonly is present in childhood and early adult life. Type II diabetes is commonly present in adulthood and is characterized by insulin resistance. There is alarm at the increasing incidence of both types in the industrialized world (2). Oxidative stress has been reported to play an important role in the development diabetic complications (3). 

Although the mechanisms underlying the alterations associated with DM are presently not well understood, hyperglycemic levels lead patients to an increased oxidative stress because the production of several reducing sugars (through glycolysis and polyol pathways) is enhanced (4). ”These reducing sugars can easily react with lipids and proteins (nonenzymatic glycation reaction) increasing the production of reactive oxygen species (ROS)” (5). Mitochondria can contribute to the development of diabetes disease because they generate a great amount of ROS (O.2) which could stimulate the progression of oxidative stress (1). ”Under normal conditions, potentially toxic ROS generated by mitochondrial respiratory metabolism are efficiently neutralized by cellular antioxidant defense mechanisms. However, this balance can easily be broken, leading to cellular dysfunction“ (6). 

On the other hand, there can be widespread disturbances of antioxidant defense systems, both enzymatic and nonenzymatic, and a reduced resistance to free radical induced tissue damage may also occur in diabetes (7). Diabetic patients have a signiﬁcant defects in antioxidant protection and adverse effects in all organic systems (8). ”Diabetes exerts a negative action on the neuroendocrine axis and hormone deficiency can enhance the action of diabetes on other organs that are dependent on the axis, for example male gonads. It is well established that low testosterone levels are related to diabetes and they can inﬂuence the morphology of reproductive accessory glands“ (9). 

DM causes many systemic complications, male infertility, impotence, retrograde ejaculation, and hypogonadism. Recently, this view has been challenged (10). “Testicular function is primarily controlled by pituitary hormones. Follicle stimulating hormone (FSH) regulates spermatogenesis, whereas luteinizing hormone (LH) controls Leydig cell function” (11). Decreases in serum levels of FSH, LH, prolactin and growth hormone have been reported in diabetes (12). “Diabetes-related effects on testicular function have been attributed to the lack of insulin. The regulatory action of this hormone is known, and observations of a direct effect on both Leydig cells and Sertoli cells have been reported” (13). 

Over the two decades, data from controlled investigations in animal models and patients have validated the therapeutic value of numerous phytotherapies for diabetes. Phytotherapies and their combinations demonstrate multiple beneficial anti-diabetic mechanisms, including modulation of carbohydrate metabolism, restoration of beta-cell integrity and function, insulin-releasing activity, improvements in glucose uptake/utilisation, antioxidant properties and a reduction in the risk of diabetic complications (14, 15). Biological antioxidants are compounds that protect biological systems against the potentially harmful effects of processes or reactions that can cause excessive oxidations (16, 17). “Most plants showed the presence of large amount of phenolics and flavonoids that have antioxidant activity” (18). 

Citrullus colocynthis had a beneficial effect on improving the glycemic profile without severe adverse effects in type II diabetic patient (19, 20). Furthermore, Al Khateeb *et al* described the physiological effects of the ethanol extract of the pulp portion of Citrullus colocynthis. The extract exhibited hypoglycemic effect on the steady state normoglycemic levels, as well as antihyperglycemic effect on steady state hyperglycaemic levels in diabetic rats. These physiological actions were mediated, at least in part, via an increase in insulin secretion (21). 

The present study was designed mainly to investigate protective effects of Citrullus colocynthis pulp in reproductive system in diabetic rats. 

## Materials and methods

This experimental study was accomplished in the Department of Biology, Faculty of Science, University of Shahid Bahonar, Kerman in fall and winter 2011. 

**Animals**

Adult Wistar male rats (weighing 230-270 gr) were used in the study. The animals were maintained under standard colony conditions with a 12 hr light/dark cycle at constant room temperature (23±2^o^C), and given adlibitum access to food and water. 

**Experimental Design**

STZ (sigma, 65/mg body wight) was used to induce diabetes. Freshly prepared STZ (dissolved in cold normal saline) was administered intraperitoneally to the rats. Three days after STZ injection, fasting serum glucose levels were measured using a Medisense Optium glucometer. Rats with blood glucose levels higher than 300 mg/dl were considered diabetics. The rats were divided into four groups (n=8):

- Group (N): Normal rats: Animals received orally normal saline for 2 wk.
- Group (D): Diabetic rats: Animals received orally by gavage normal saline for 2 wk.
- Group (N+C): Received orally Citrullus colocynthis pulp powder 10mg/kg.bw dissolved in normal saline for 2 wk.
- Group (D+C): Diabetic rats received orally Citrullus colocynthis pulp powder 10 mg/kg.bw dissolved in normal saline for 2 wk.

The animals were sacrified on the 14^th^ day of the experiment first deeply anesthetized with CO_2_, and then assassinated by giyotin. The testes and epididymis were removed immediately and prepared for oxidant and antioxidant assays.

**Catalase (CAT) and peroxidase (POD) assay**

Tissues were homogenized in 50 mM phosphate bufer (pH=7.4). The homogenate was centrifuged at 10000 gr for 10 min at 4^o^C. CAT activity was measured by the method of Aebi (22). To a cuvette containing 1.5 ml of catalase mixture (H_2_O_2_+50 mM phosphate bufer), the 100µl tissue supernatant was added. The reaction was started by decomposition of H_2_O_2_ and CAT activity was measured spectrophotometrically at 240 nm. POD assay: POD activity of tissues was measured by the method of Plewa *et al *(23). To a cuvette containing 2.5 ml of POD mixture (H_2_O_2_+50 mM phosphate bufer+ guayacol), 20µl of tissue supernatant was added. The reaction was started by the oxidation of guayacol and POD activity was measured spectrophotometrically at 470 nm.

**MDA assay**

Thiobarbituric Acid Reactive Substances (TBARS) level, measured as an index of malondialdehyde production and hence lipid peroxidation, were assessed in the tissues by the method of Heath and Packer (24). In brief, tissue supernatant (1 ml) was added to test tubes containing 4 ml of TCA 20% (Trichloroacetic acid) containing TBA 0.5% (Thiobarbituric acid) and the reaction mixture was heated at 95^o^C for 30 min and after cooling, centrifuged at 10000 gr for 10 min and MDA-TBA complex was measured spectrophotometrically at 532 nm. 

**H**_2_ **O**_2_ **assay**

H_2_O_2_ level measured as an index of oxidant factors, was assessed in the tissues by the method of Velikova *et al *(25). Tissues (0.1 gr) were homogenized in 1 ml TCA (pH=7.4). The homogenate was centrifuged at 10000 gr for 10 min at 4^o^C. H_2_O_2_ concentration of tissue was measured in a cuvette containing 0.5 ml of tissue supernatant and 0.5 ml phosphate buer 10 mM (pH=7.4) and 1ml of potasium Iodid 1 mM was added and H_2_O_2_ concentration measured spectrophotometrically at 390 nm. 

**Ethical consideration**

The study protocol and all animal procedures were approved by the research committee of Kerman University of Medical Sciences (Animal code: Ir.kmu.rec.1395.237). 

**Statistical analysis**

Data were expressed as mean±SEM. Statistical differences between the groups were analyzed by using the one-way analysis of variance (ANOVA) test and TUKEY post test with SPSS version 18. P<0.05 was considered significant.

## Results

**Figure 1:**
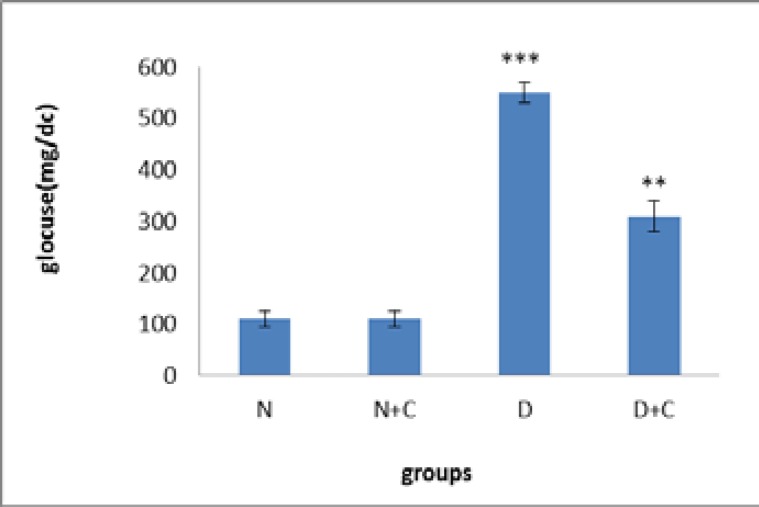
Glucose concentration in experimental groups. n=8, Mean ± SEM. ** Significant difference (P<0.01) with N, N+C and D groups. *** Significant difference (p<0.001) with N and N+C groups

**Figure 2:**
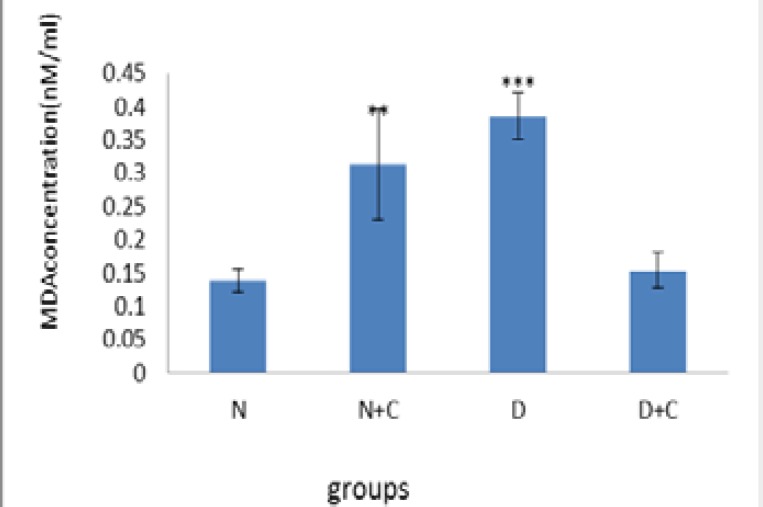
MDA concentration in testis in experimental groups. n=8, Mean ± SEM. ** Significant difference (p<0.01) with N, D and D+C groups. *** Significant difference (p<0.001) with N and D+C groups

Figure 1 shows that glucose concentration in D and D+C groups is significantly higher compared to N and N+C groups (p<0.001 and p<0.01, respectively). Also D+C group showed a significant decrease compared to D group (p<0.01). Figure 2 shows that MDA concentration in testis in N+C group is significantly higher compared to N and D+C groups and lower compared to D group (p<0.01). D group showed a significant increase compared to N and D+C groups (p<0.001).

**Figure 3:**
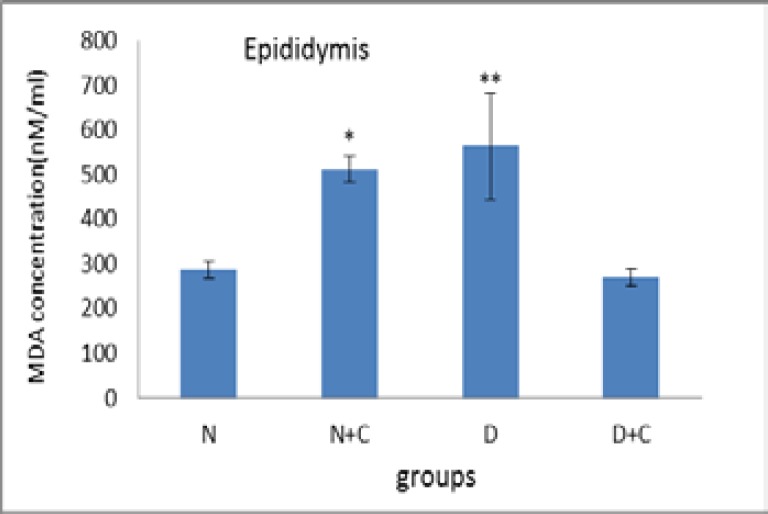
MDA concentration in epididymis in experimental groups. n=8, Mean ± SEM. * Significant difference (p<0.5) with N and D+C groups. ** Significant difference (p<0.01) with N and D+C groups

**Figure 4:**
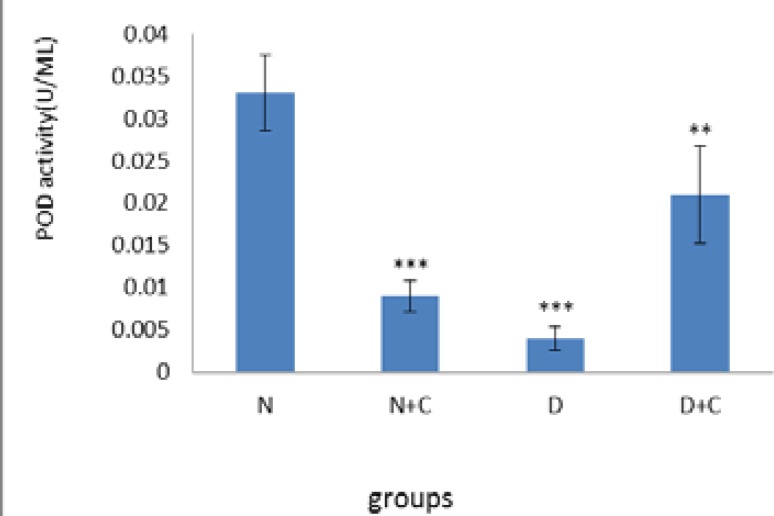
POD activity in testis in experimental groups. . n=8, Mean ± SEM. ** Significant difference (p<0.01) with N, N+C and D groups. *** Significant difference (p<0.001) with N group

Figure 3 shows that MDA concentration in epididymis in N+C and D groups is significantly higher compared to N and D+C groups (p<0.05 and p<0.01 respectively). Figure 4 shows that POD activity in testes in N+C and D groups significantly decreased compared to N group (p<0.001). Also D+C group showed a significant increase compared to D and N+C groups and a significant decrease compared to N group (p<0.01).

**Figure 5:**
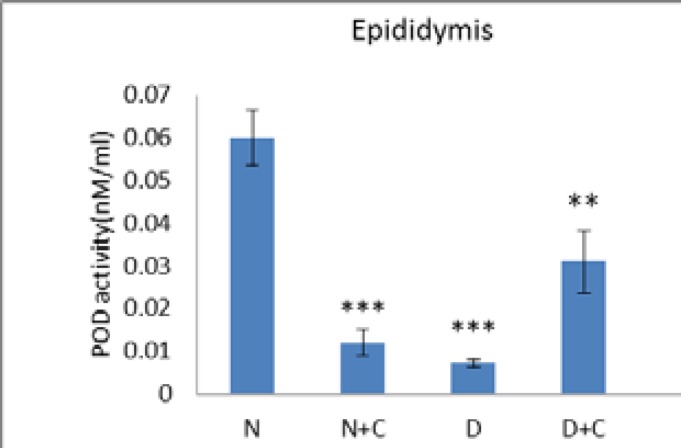
POD activity epididymis in experimental groups. n=8, Mean ± SEM. ** Significant difference with N, N+C and D groups. *** Significant difference with N group

**Figure 6:**
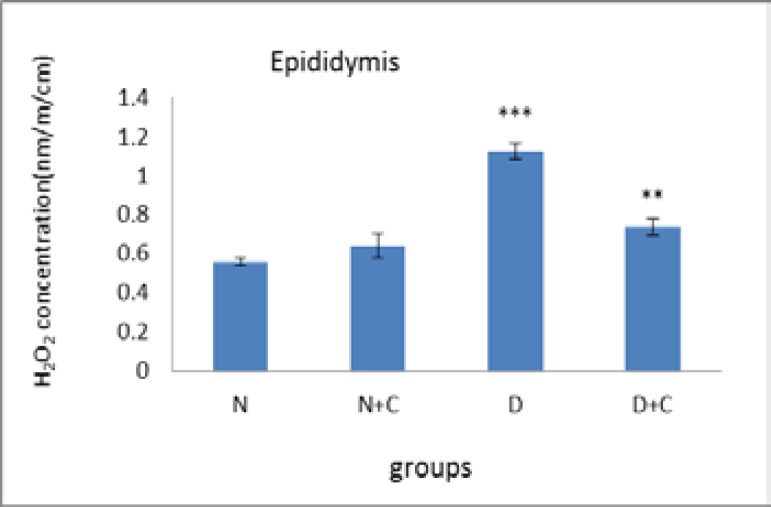
H_2_O_2_ concentration in epididymis in experimental groups. n=8, Mean ± SEM. ** Significant difference (p<0.01) with D group. *** Significant difference (p<0.001) with N and N+C groups

Figure 5 shows that POD activity in epididymis in N+C and D groups is significant decreased compared to N group (p<0.001). Also D+C group showed a significant increase compared to D and N+C groups and a significant decrease compared to N group (p<0.01). Figure 6 shows that H_2_O_2_ concentration in epididymis in D group is significantly higher compared to N and N+C groups (p<0.001). Also D+C group showed a significant decrease compared to D group (p<0.01).

**Figure 7:**
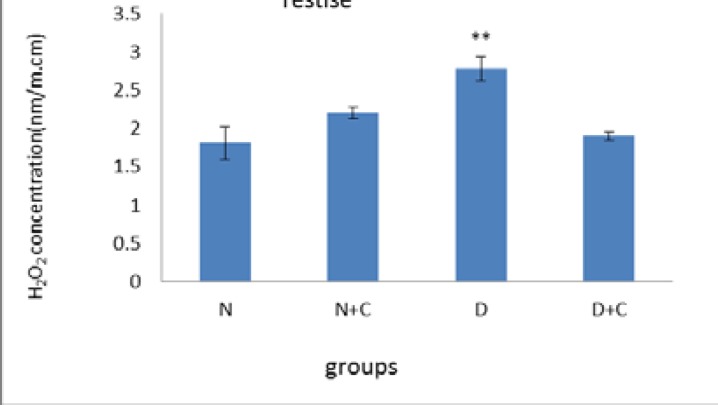
H_2_O_2_ concentration in testis in experimental groups. n=8, Mean ± SEM. ** Significant difference (p<0.01) with N, N+C and D+C groups

**Figure 8:**
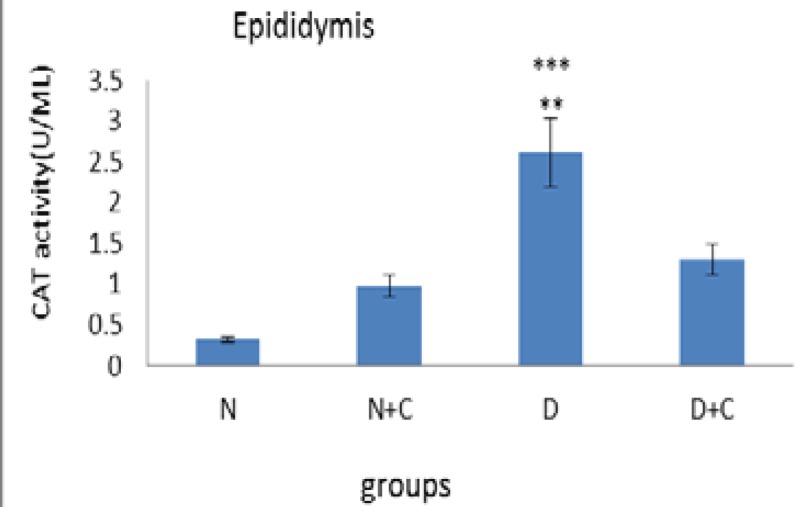
CAT activity epididymis in experimental groups. n=8, Mean ± SEM. ** Significant difference (p<0.01) with N+C and D+C groups. *** Significant difference (p<0.001) with N groups

**Figure 9:**
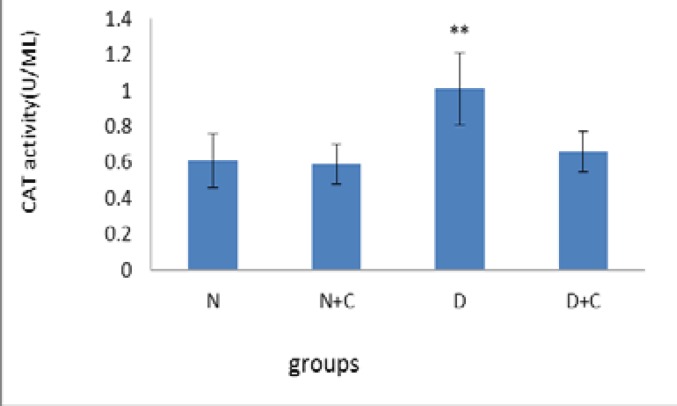
CAT activity testis in experimental groups. n=8, Mean ± SEM. ** Significant difference (p<0.01) with N, N+C and D+C groups.

Figure 7 shows that H_2_O_2_ concentration in testis in D group is significantly higher compared to N, N+C and D+C groups (p<0.01). Figure 8 shows that CAT activity in epididymis in D group is significantly higher compared to N group (p<0.001) and N+C and D+C groups (p<0.01). Figure 9 shows that CAT activity in testis in D group is significantly higher compared to N, N+C and D+C groups (p<0.01).

## Discussion

Diabetes mellitus is a life threatening metabolic disorder and it is estimated that its annual incidence rate will continue to increase in the future worldwide (22). Increased oxidative stress is widely accepted to be the main factor playing a role in the development and progression of diabetes and its complication (23, 6). 

Traditional medicinal plants are used throughout the world for a range of diabetic complications. Citrullus colocynthis pulp has the antioxidant and antidiabetic compounds and is effective in reduction of oxidative stress induced by DM (24, 25). Results of the present study showed that treatment of diabetic rats with Citrullus colocynthis pulp decreased oxidant factors and support antioxidant factors in the reproductive system in diabetic rats. These changes result in diminishing reproductive complications due to diabetes.

In the present study, we showed that diabetic group had a significant increase in MDA levels in testes and epididymis compared to normal group. MDA level has been widely used as a marker of lipid peroxidation in cell and body fluids in both clinical and experimental studies (26). The increase in lipid peroxidation associated with the decrease in antioxidative defense (3). Lipid peroxidation is considered a hallmark of oxidative stress, in which ROS interact with polyunsaturated fatty acids, and lead to the formation of products such as MDA and 4-HNE (4-hydroxynonenal), which then results in damages to the membrane components of the cell, cell necrosis and inﬂammation (27).

The aldehydic products of lipid peroxidation such as MDA and 4-HNE are more cytotoxic and stable than ROS and react quickly with cellular constituents (28). Besides, Halliwel *et al* showed that MDA and 4-HNE are modulators of signal transduction pathways that disturb cellular activities (29). H_2_O_2_ concentration in testes and epididymis tissues in diabetic group had a significant increase compared to normal group that was similar to observation by Bary *et al* (30). One mechanism is that insulin deficiency, promotes beta oxidation of fatty acids, resulting in H_2_O_2_ formation (31). We observed that the diabetic group had a significant decrease in POD and an increase in CAT activity in testis and epididymis compared to normal group. Our result is in agreement with the study of Searle *et al* (32). 

POD and CAT are two of the main defenses against oxidative injuries. CAT is present in the peroxisomes of nearly all aerobic cells but not present in the mitochondria (33). There are inconsistence reports in the level of CAT activity in diabetic animals and humans. For example, CAT activity is consistently found to be elevated in heart and aorta, as well as brain of diabetic rats. In contrast to decreased CAT activity in lung, hepatic and red blood cell, this activity in liver and renal of diabetic animals is increased (34). It is suggested that the increased CAT activity reﬂects the increased production of H_2_O_2_, which agrees with the ﬁndings of other observations (35, 36). 

Also, we think that the deference in tissue CAT activities may be attributed to the differences in tissue antioxidant capacity and the severity of oxidative stress among the organs (37). In this regard, similar tissue-speciﬁc changes in H_2_O_2_ and detoxifying enzyme have been reported by Missirya and Gindy in diabetic rats (36). Moreover, the decrease in POD activity could be the result of a reduced synthesis of these enzyme protein as a result of higher accumulation of free radicals, as reported by Halliwell *et al* (38). 

Diabetic rats treated with Citrullus colocynthis showed that the levels of MDA and H_2_O_2_ and CAT activity significantly decreased and POD activity significantly increased in the testis and epididymis compared to the diabetic rats. This change indicates that Citrullus colocynthis pulp has antioxidant capacity and causes reduced lipid peroxidation reduction. Our results are consistent with the study of Canada *et al* (39). Citrullus colocynthis has free radicals cleansing capacity because of effective action against pathological alterations caused by the superoxide and H_2_O_2_ (32).

It was showed that phenolic compounds isolated from Citrullus are of great interest due to their antioxidative and anticarcinogenic activity. They play very important roles in absorbing and neutralizing free radicals. They contain not only minerals and primary metabolites, but also a diverse array of secondary metabolite with antioxidant potential (40). One study also revealed that different Citrullus colocynthis extracts have an insulinotropic effect which could at least partially account for the antidiabetic of this plant (41). Therefore, it is noteworthy that at antioxidant and antidiabetic properties of compound Citrullus collocynthis pulp, suggested that using this plant pulp has a direct and indirect therapeutic effects to reduce side effects of diabetes induced oxidative stress in the present study.

It is suggested that Citrullus colocynthis pulp, due to its antioxidant and antidiabetic compounds, has both direct and indirect effects in reducing oxidative stress induced by DM. Direct effect of the pulp is resulted from the phenolic compounds with antioxidant properties which neutralize and eliminate oxidant factors with several mechanisms (24). The indirect effects include pancreatic β-cell activation, due to active components such as saponozoyidhes, which causes increased insulin secretion and this in turn leads to glucose utilization. It has been established that decreased glucose level reduces non-enzymatic oxidation, glycosylation, polyol and hexosamine pathway, activity of protein kinase C, and ultimately leads to the reduction of oxidative stress (32).

The results of the present study showed that Citrullus colocynthis pulp administered to normal rats (N+C) increased H_2_O_2_ and MDA concentration and CAT activity and decreased POD activity compared to the normal group, and these results are consistent with the study of Shivakumar, in which administration of the plant’s pulp increased oxidative damage in normal rats. Citrullus colocynthis extract stimulates formation of H_2_O_2_-induced production of free radicals and lipid peroxidation cause tissue damage (20).

## Conclusion

It is concluded that treatment of diabetic rats with Citrullus colocynthis pulp decreased oxidant factors and support antioxidant factors in the testis and epididymis in diabetic rats. These changes result in improved reproductive complications due to diabetes.

## Note

***This article extracted from M.Sc. thesis. (Fereshteh Ostovan)***
